# Diet in Disease

**Published:** 1893-07-22

**Authors:** Ernest Hart

**Affiliations:** Bachelier-ès-Sciences-es-Lettres (rèstreint), formerly Student of the Faculty of Medicine of Paris, and of the London School of Medicine for Women


### Diet in Disease.

By Mrs. Ernest Hart, Bachelier-ès-Sciences-es-Lettres (rèstreint), formerly Student of the Faculty of Medicine of Paris, and of the London School of Medicine for Women.

### XXVI.—On Thinning and Fattening.

*(Continued.)*

There is no doubt that in spite of the controlling influence of diet some persons have a tendency to grow fat, and that others either become or remain thin. It is more difficult to make a person thin by nature grow fat, than to reduce a fat person. The articles of food—the butter, cream, puddings, and sweets—which are eliminated from the dietary of the fat may be greatly increased in the dietary of the thin, often without making any marked difference in their condition and weight. By a strange perversity of nature also the thin person frequently dislikes the food that would make him grow fat. It is probable, moreover, that the greater activity of the spare body keeps it thin.

When, however, thinness becomes progressive, and approaches emaciation, and is moreover associated with anæmia, weakness, and that group of nervous symptoms known under the name of hysteria, it may be very successfully treated as a symptom of a disease of nutrition.

The system of treatment consists in rest, isolation, over-feeding, passive exercise or massage, and electricity, and is known by the name of its founder, Dr. Weir-Mitchell. The patient is taken from home and strictly isolated from friends and family; she is put to bed, and in extreme cases is not even allowed to sit up in bed, but is fed by a nurse. The patient should be weighed before being put to bed, and should be weighed at frequent intervals during the treatment. She is first placed on a milk diet, and for the first day or two from three to four ounces of milk are given every two hours. The milk may be slightly warmed, and if it is particularly distasteful to the patient it may be flavoured with a little tea or coffee. The quantity of milk taken is gradually increased, and the intervals lengthened to three hours, till at last two quarts are taken in the twenty-four hours. This rest in bed and the simple milk diet "nearly always dismisses," says Dr. Weir-Mitchell, "as if by magic, all the dyspeptic conditions" from which the patient had previously suffered. The circulation is at the same time stimulated and the muscles undergo passive exercise by being kneaded by massage and moved by electric currents. The bowels are carefully regulated. After from four to seven days a little solid food is taken, namely, bread and butter for breakfast, and a milk pudding for dinner. A day or two later, fish and chicken or a mutton chop are added, first either at the mid-day or evening meal, and then at both. In about ten days the patient is put on three full meals daily, and the diet will be as follows:—

Milk, sixty to eighty ounces.
Breakfast, porridge and cream.
Second Breakfast, cocoa and egg, bread and butter.
Luncheon, fish, bread, pudding, and milk, or chicken, vegetables, and pudding.
Dinner, mutton or beef, two or three kinds of vegetables, milk pudding, or stewed fruit with cream.

Extract of malt may be given with one or more of the supplies of milk, and in some cases cod liver oil is also prescribed.

Dr. Weir-Mitchell says of this treatment: "No troublesome symptoms usually result from this full feeding, and the patient may be made to eat more largely by being fed by her attendant;" and as to the beneficial effect, he says, "I have watched again and again, with growing surprise, some listless, feeble, white-blooded creature learning by degrees to consume these large rations, and gathering under their use flesh, colour, and wholesomeness of mind." Tonics are also given in rather full doses, iron, arsenic, and sulphate of strychnine. Where there is no alcoholic habit to break, a small daily dose of whisky or wine is found to assist in the increase of fat. As the diet is increased and maintained at its highest point, it is necessary to carefully watch the urine, and if an excess of urates is found, it is an indication that the amount of food must be reduced.

At the end of five or six weeks the patient will be found to have gained considerably in weight and strength, and the muscles to have become firmer and fuller. The massage can then be decreased, and normal exercise on foot allowed. The excessive diet is also slowly reduced; the quantity of the milk is first reduced, then the intermediate meals are dropped, and gradually the patient returns to a normal, active, open-air life. The cure may be completed by a sea voyage or a foreign tour, and the patient, once restored to health, family, and to a life of interest and usefulness, rarely relapses. The following cases will illustrate the treatment and its results:—

### Under Dr. Weir-Mitchell.

Mrs. C——. Kept in bed and fed by an attendant.

First Day.—One quart of milk in divided doses every two hours.

Second Day.—Cup of coffee on waking. Two quarts of milk in divided portions every two hours.

Third to Sixth Day.—Same diet.

Seventh, Eighth, and Ninth Days.—Same diet, with a pint of raw soup in three portions.

Tenth Day.—7 a.m., coffee; 7.30, half-pint of milk; 10 a.m., ditto; noon, 2, 4, 6, 8, and 10 p.m., ditto; soup at 11 a.m. and 5 and 9 p.m.

Fourteenth Day.—Egg and bread and butter added.

Sixteenth Day.—Dinner added and iron.

Nineteenth Day.—The entire diet was as follows: 7 a.m., coffee; 8 a.m., iron and malt extract; breakfast, consisting of a chop, bread and butter, a tumbler and a half of milk; 11 a.m., soup; 2 p.m., iron and malt; dinner of anything she liked, with 6 oz. of Burgundy or dry champagne, and at the end one or two tumblers of milk; 4 p.m., soup; 7 p.m., malt, iron, bread and butter, usually some fruit, commonly two glasses of milk; 9 p.m., soup.

(At 12 noon massage for an hour; at 4 30 p.m. electricity applied for an hour.)

At sixth week soup and wine were dropped, iron lessened one half, massage and electricity only on alternate days.

At ninth week milk reduced to a quart. All mechanical treatment ceased.

*Result.*—Gain in flesh about face in second week. Weight rose in two months from 96 to 136 lbs.; gain in colour equally marked. At ninth week drove out. Cure complete and permanent.

### Under Dr. Playfair.

A. B., aged 32. Rest in bed, isolation.

First Day.—22 ozs. of milk in divided doses.

Second Day.—50 ozs. of milk in divided doses.

Third Day.—50 ozs. of milk in divided doses. Massage half an hour.

Fourth Day.—50 ozs. of milk in divided doses; egg and bread and butter; 40 minims of dialysed iron in two doses. Massage 1½ hours.

Eighth day.—Fifty ounces of milk in divided doses, mutton chop, porridge, and a gill of cream; maltine twice daily. Massage three hours, electricity half an hour; continued to end of treatment.

Fifteenth day.—Three full meals daily of fish, meat, vegetables, cream, and fruit; two quarts of milk, and two glasses of Burgundy.

Twenty-second day.—Amount of food lessened.

*Result.*—On twenty-second day sat in a chair for an hour; after a month walked downstairs, and went for a drive. Enormous increase in size. Cure complete and permanent.

The success of this treatment is now established. Dr. Weir-Mitchell is anxious to insist on the fact that increase of weight should correspond with increased richness of blood. The number and colour of the red corpuscles of the blood should be examined and estimated during treatment. This is easily accomplished by means of the delicate instruments designed by both Malassez and Gowers for counting the number of red corpuscles in a minute drop of blood, and estimating the amount of hæmaglobin they contain. Dr. Weir-Mitchell insists that there is an intimate association between the gain and loss of fat and the gain and loss of red blood corpuscles. He therefore finds those cases in which these usual conditions are reversed, when there is an excessive deposit of fat with a decreased number of red blood corpuscles, very intractable and difficult to treat. He recommends that in such cases the patient should be put to bed, massage should be freely used, and the diet restricted to skimmed milk, or to milk and broth free from fat. When the weight is lowered iron should be freely given, and by degrees a general diet. The red blood corpuscles will be found under this treatment to have increased in number, and as the weight diminishes strength increases, and health is re-established.

Dr. Weir-Mitchell gives a case of a lady, aged 45, 5 ft. 4½ in. high, who weighed 190 lb. (13st. 4lb.). She was anæmic, feeble, and breathless. "She was kept in bed for five weeks. Massage was used at first once daily, and after a fortnight twice a day, while milk was given, and in a week made the exclusive diet. Her average loss for thirty days was a pound a day, and the diet was varied by the addition of broth after the third week, so as to keep the reduction within safe limits. . . . After two weeks I gave her the lactate of iron every three hours in full doses. On the fourth week additions were made to her diet list, and Swedish movements were added to massage, which was applied but once a day; and during the fifth week she began to sit up and move about. Her weight at the seventh week had fallen to 145 lbs., and her appearance has decidedly improved. . . . Now, after two years, she is a well and vigorous woman."

This case illustrates the fact that each case should be treated scientifically, intelligently, and on its merits. It is as futile as it is dangerous for the unlearned to prescribe dietetic rules for the ailing, the weak, and the obese. "She wants support," a sympathetic friend will say of a poor invalid suffering from the nausea and weakness of advanced Bright's disease, and will proceed to recommend steak and port wine, ignorant of the fact that she is thereby increasing the effete products in the blood of the patient, which are the causes of the symptoms remarked. Or another will tell of a great reduction in size brought about in himself by a meat diet and mountaineering, and will unhesitatingly prescribe the same for a fat friend whose pallor and lethargy bespeak a fatty heart and perhaps damaged kidneys. Dietetic treatment is but reasonable medical treatment based on principles or knowledge more scientific and accurate as a rule than that of drugs, and should be undertaken or prescribed after a careful study of the causes of the condition complained of.

